# Data of food challenges of an outpatients’ pediatric allergy clinic in the Mediterranean island of Crete, Greece

**DOI:** 10.1186/2045-7022-5-S3-P95

**Published:** 2015-03-30

**Authors:** Evangelia Stefanaki, Georgia Chatzitzanou, Maria Anatoliotaki, Sofia Stefanaki, Vassiliki Aggelakou

**Affiliations:** 1 Venizelion General Hospital, Heraklion, Crete, Greece

## Introduction

Food allergy is defined as an adverse immunologic response to a dietary protein. Diagnosis involves a careful history and diagnostic tests, such as skin prick testing, serum-specific immunoglobulin E (IgE) testing and, if indicated, oral food challenge which is the gold standard of diagnosis.

## Methods

We studied retrospectively the food challenges organized by our Outpatients Paediatric Allergy Clinic between 2011-2013 and we recorded the culprit food allergens, age at challenge, challenge outcome, diagnostic work-up before challenge and coexistence of eczema.

## Results

We recorded 38 children that underwent 75 challenges. The median age at challenge was 15 months and 68,4% (26/38) of the children were boys. 50% (14/28) of the children had a family history of atopy. 66 % (25/38) of the children were diagnosed with atopic dermatitis at the time of reaction or/and at diagnosis. 73% (19/26) of the children with atopic dermatitis had only mild persistent signs of the disease. 68,4%(26/38) of all the children had a history of clinical reaction (in 77%,20/26 of the patients the reaction was immediate) and the median age at first reaction was 6 months while 31,6% (12/38) were only sensitized to the allergen of the challenge. 52,6% (20/38) were sensitised or reacted to only one allergen most commonly to egg (6/20) and milk (6/20). We recorded 27 (36%) challenges to milk- 7 to partially hydrolysed milk (after negative prick-to-prick PTP test to pHA milk), 10 to evaporated milk, 4 to common formula, 3 to cake with milk, 2 to fresh milk and 1 to yoghurt. At the time of milk challenges the average diameter for prick to prick test was 5,1 mm for HA, 3,6mm for evaporated milk and 7,3 mm for cake only with milk. We also recorded 22 (29,7%) challenges to egg (7/22 to cake with egg). The average PTP was 1,8 mm to boiled egg white and 0,84 mm for egg yolk. We finally recorded 6 challenges to fish, 6 to wheat, 2 to lentils and 1 to potato. Only 1/75 challenges was positive and the reaction was only mild.

## Conclusions

Our food challenges under safe conditions placed no patient at risk for serious reactions. Atopic dermatitis although mild is still a major risk factor for food allergy. Different types of milk fit differently to each milk allergic patient.

